# 2137. Real World Experience with Eravacycline for the Treatment of Infections Caused by Carbapenem-Resistant *Acinetobacter baumannii*

**DOI:** 10.1093/ofid/ofad500.1760

**Published:** 2023-11-27

**Authors:** Andy Lim, Yi Guo, Terrence D McSweeney

**Affiliations:** Montefiore Medical Center, Flushing, New York; Montefiore Medical Center, Flushing, New York; Montefiore Medical Center, Flushing, New York

## Abstract

**Background:**

Carbapenem-resistant *Acinetobacter baumannii* (CRAB) are considered an urgent public health threat responsible for causing severe infections with high associated mortality. CRAB isolates are often resistant to several antibiotic classes thereby limiting treatment options. Eravacycline is a novel tetracycline antibiotic with potent *in vitro* activity against CRAB. Per the recent Infectious Diseases Society of America (IDSA) Guidance on the Treatment of Antimicrobial-Resistant Gram-Negative Infections, clinical data are limited for eravacycline use for CRAB infections. The purpose of this study was to evaluate clinical outcomes of patients treated with eravacycline for CRAB infections at Montefiore Medical Center.

**Methods:**

This was a retrospective chart review of patients 18 years of age and older who received eravacycline for at least 72 hours for the treatment of an infection due to CRAB between January 1, 2019 to February 28, 2023. Patients were excluded if they had a urinary tract infection. Patient demographics, risk factors, type/source of infection, clinical outcomes, etc. were collected.

**Results:**

Eighteen patients received eravacycline for the treatment of non-urinary CRAB infections (Table 1). Ventilator-associated pneumonia was the predominant infection, representing 14/18 (78%) cases. High rates of antibiotic non-susceptibility were demonstrated in all cases. Median eravacycline minimum inhibitory concentration (MIC) was 1 mg/L, with a range of 0.19 – 6 mg/L. Median duration of eravacycline therapy was 7 days. Overall, the clinical success rate was 33% (6/18). Four patients (22%) experienced progression or worsening of symptoms that required a change in antibiotics. Of 11 evaluable patients, three patients (27%) had a recurrent infection caused by CRAB within 30 days of completion of eravacycline therapy. All-cause 30-day mortality was 56% (10/18), with six deaths (33%) attributed to CRAB infection.

**Figure:**
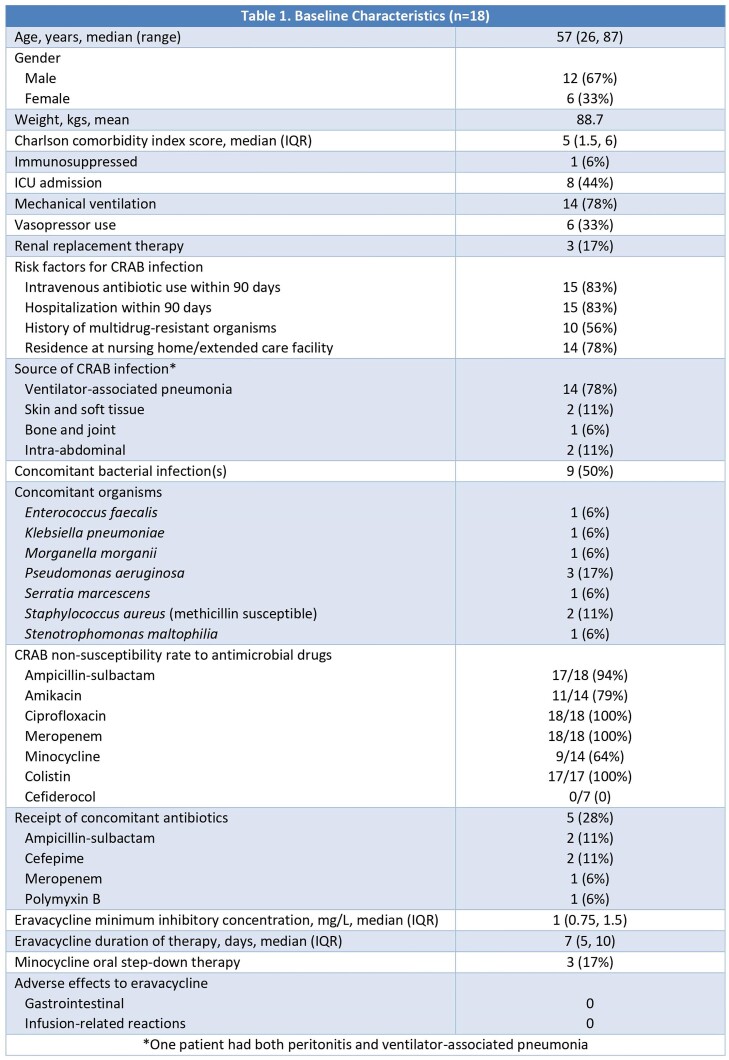

**Figure:**
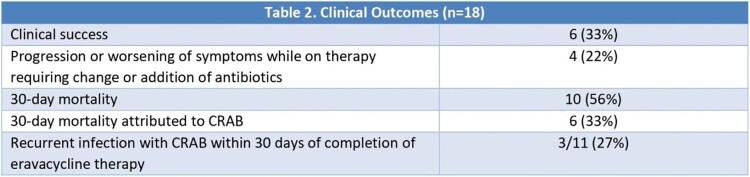

**Conclusion:**

CRAB infections pose a significant challenge in the healthcare setting and are associated with high mortality rates. Eravacycline remains a last resort option for these difficult to treat infections. Further study is warranted for eravacycline use for CRAB infections.

**Disclosures:**

**All Authors**: No reported disclosures

